# Semantics of European poetry is shaped by conservative forces: The relationship between poetic meter and meaning in accentual-syllabic verse

**DOI:** 10.1371/journal.pone.0266556

**Published:** 2022-04-12

**Authors:** Artjoms Šeļa, Petr Plecháč, Alie Lassche

**Affiliations:** 1 Institute of Polish Language, Polish Academy of Sciences, Krakow, Poland; 2 University of Tartu, Tartu, Estonia; 3 Institute of Czech Literature, Czech Academy of Sciences, Prague, Czechia; 4 Leiden University, Leiden, Netherlands; University of Birmingham, UNITED KINGDOM

## Abstract

Recent advances in cultural analytics and large-scale computational studies of art, literature and film often show that long-term change in the features of artistic works happens gradually. These findings suggest that conservative forces that shape creative domains might be underestimated. To this end, we provide the first large-scale formal evidence of the association between poetic meter and semantics in 18-19th century European literatures, using Czech, German and Russian collections with additional data from English poetry and early modern Dutch songs. Our study traces this association through a series of unsupervised classifications using the abstracted semantic features of poems that are inferred for individual texts with the aid of topic modeling. Topics alone enable recognition of the meters in each observed language, as may be seen from the same-meter samples clustering together (median Adjusted Rand Index between 0.48 and 1 across traditions). In addition, this study shows that the strength of the association between form and meaning tends to decrease over time. This may reflect a shift in aesthetic conventions between the 18th and 19th centuries as individual innovation was increasingly favored in literature. Despite this decline, it remains possible to recognize semantics of the meters from past or future, which suggests the continuity in meter-meaning relationships while also revealing the historical variability of conditions across languages. This paper argues that distinct metrical forms, which are often copied in a language over centuries, also maintain long-term semantic inertia in poetry. Our findings highlight the role of the formal features of cultural items in influencing the pace and shape of cultural evolution.

## Introduction

Recent advances in cultural analytics [1] and large-scale computational studies of creative domains such as art, literature and film provide increasing evidence that change in features of artistic works happen gradually over extended periods of time. This can be seen in lexical choices in fiction [2, 3], writing styles [4, 5], the shortening of cinematic shot lengths [6], the long-term recognizability of literary genres [7] and aesthetic choices in poetry [8]. This picture is puzzling because it suggests continuity and slow global processes in traditions that scholars have often perceived as volatile fields of innovation, competition and constant conflict of elites [9, 10]. The contrary evidence for a punctuated equilibrium pattern of cultural evolution [11] or a cyclical turn-around of styles [12–14] is also abundant, but a question remains: Are we underestimating the continuity and conservative forces at work in cultural traditions associated with creative freedom? This study asks this question about a practice which tends to be imagined as extremely individualistic: the composition of poetry. We apply a data-driven semantic analysis to the formal characteristics of texts across several languages. Our goal is to address one of the fundamental issues in versification studies: the relationship between poetic form and its meaning.

Modern poetry is often seen as a space of boundless innovation and individualized self-expression. However, there is at least one aspect of poetry that is defined by conservative persistence over centuries and millennia: poetic meter (Table 1). Meters are rarely invented individually; rather they are stable prosodic patterns that usually arrive in the hands of a poet after having long histories of use in local and global traditions. This persistence of formal features in poetry invites us to shift our focus from individuals to meters as cultural items that participate in long transmission chains [15] that diffuse them far and wide.

**Table 1:** Examples of accentual-syllabic metrical types and labeling strategies. S—denotes a strong position in the foot (stress expected), W—weak.

Meter	Foot	Pattern	Metrical Type	Label
Iamb	WS	WS|WS|WS|WS|WS Thus was|I, slee|ping, by | a bro|ther’s hand Of life,| of crown,| of queen,| at once| dispatch’d	iambic pentameter	I5
Trochee	SW	SW|SW|SW|S(W) Tell me| not in| mournful | numbers, Life is | but an | empty | dream	trochaic tetrameter	T4
Dactyl	SWW	SWW|SWW|SWW|S(WW) Brightest and| best of the | sons of the| morning	dactylic tetrameter	D4
Amphibrach	WSW	WSW|WSW|WSW|WS(W) Oh, hush thee,| my baby|, thy sire was | a knight Thy mother | a lady| both lovely | and bright	amphibrachic tetrameter	A4
Anapest	WWS	WWS|WWS He is gone | on the moun|tain He is lost | to the for|est	anapestic dimeter	An2

Research into historical poetics and metrics strongly suggests that meter is not agnostic to meaning [16–19]. Usage of meters is affected by what was previously written in these meters and by additional signals that poetic forms accumulate and carry over time. In oral traditions, a difference in meter was often functional: it supported genre diversification between the poles of epic and lyric poetry [20]. The rise of written and printed media allowed more diverse poetic metrical forms and expanded their sources. New forms could emerge from the standardization and restructuring of cultural borrowings: foreign traditions, classical Latin and Greek examples, adaptations of local oral versification systems. Through their usage, mnemonic capacities and generic conventions, metrical forms allegedly maintained fuzzy but distinct traditions of usage that were reproduced and updated by generations of poets. This theory of a relationship between a meter and its meaning is known as the “semantic halo of meter”.

In this study, we aim to computationally test the presence of the semantic halo across several modern European poetic traditions (18–20th c.). Most of the evidence for a semantic halo comes from sporadic informal studies of a few European (mostly Slavic) literatures [17, 19–22]. Several recent attempts to formalize this concept have shown lexical differences between metrical forms in individual traditions [23, 24] while also broadly confirming the presence of the semantic halo in 18-to-20th century Russian verse [25]. This growing body of evidence suggests that the mechanism which binds form and meaning is widespread and could potentially be universal. However, a reliance on close reading, a lack of formalization and the sporadic nature of the research to date have limited scholars’ ability to generalize about the semantic halo, its nature and historical dynamics.

The current study relies on several assumptions about the nature of poetic form and its relationship to verse semantics. First, we assume that poetry is a socially learned practice that is subject to cultural evolutionary forces [26–28]. Poetic formal features such as meter and rhyme are reproduced and developed through a copying process that may be influenced by various factors, or biases. For example, acquisition and popularity of meters might depend on their intrinsic features, or fit to a language prosody (stress-timed languages often adopt accentual-syllabic meters). It also might be driven by cultural prestige, like medieval and early modern Latin hexameter: despite not perceiving any distinction between long and short vowels anymore, scholars and students continued to write “correct” quantitative hexameters for centuries, relying solely on memorized rules [20].

Second, we assume that the meter and meaning association is mainly dependent on its historical environment—the ways it was copied and transmitted before. Despite increasing evidence for the role of iconicity and non-arbitrariness of form-meaning association in natural languages [29], available research on meters and their modern histories do not suggest that the relationships between poetic meter and meaning are fundamentally iconic (but these claims do exist [22]). Usage of a meter may differ across closely-related languages and cultures, can shift over time, be intentionally subverted and influenced by single individuals. It is also hard to defend any perceptual or motor foundation for the analogy between poetic form and meaning: even in special cases like work songs or sea shanties where rhythm organizes movement and action, the iconic connection is firstly established between form and function, not between form and semantics. If an alien would hear a recording of a sea shanty that did not use any maritime themes (which is common), liked it and started imitating this particular recording, the history of this form in the alien’s own tradition may very well lack any mentions of, or associations with the sea.

The halo effect, however, does resemble systematicity in languages. Systematicity describes non-random differences in word forms across abstract categories, like word classes or parts of speech [30, 31]: form regularities here provide cues to facilitate class distinction and learning. With poetic meters the relationship might be seen as reversed: many individual poems exhibit semantic regularities that map onto a limited amount of most frequent prosodic forms. Similarly to the documented emergence of systematicity in iterative learning experiments [32, 33], it is plausible that the distinct metrical patterns emerged in oral traditions to maximize semantic and structural differences in response to cognitive and environmental factors (e.g. aiding the memorization and recreation of different texts [34]). Treating meter-meaning relationship as systematic might provide support for claims of non-arbitrariness cases in semantic halo, like association between length of poetic line and mood, widespread in Europe (longer meters tend to be serious and convey importance, while shorter meters associate with lighter themes, songs and jokes) [17].

Given the lack of more detailed research in the domain, we look at the semantic halo in modern traditions as dependent on the power of structural differences and exposure to previous examples. In theory, nothing should stop a poet from choosing any theme when employing meter X, but in practice, poets often use meters somewhat similarly to how they were used in the past. The theory of semantic halo implies that meaning is copied preferentially within metrical forms, not across them. This might be partly explained by systematicity, since modern poetry inherited forms that were made different before: they did sound different, emerged from various sources [20], cast different systematic constrains on morphology and syntax [35] that guided the reproduction of syntactic formulas and provided mnemonic cues to a reader or a poet. Even under highly individualistic aesthetics, it would be hard to overcome the power of poetic meters and use them completely free. Indeed, to be “free” of a meter is to get rid of meters altogether, which is what happened in many Western traditions during the course of the 20th century.

Social transmission factors then might be seen as responsible for introducing variation to the grip that meters have on poets. For example, we might assume that at the population level poets are driven by a conformity bias and copy the most frequent examples of meter usage. Normative expectations were frequently taught explicitly, indicating appropriate meters for the occasion (like alexandrine verse for classic French tragedy, or iambic tetrameter for Russian or German ode). However, the pressure for conformity is unlikely to be constant across time: traditional scholarly narrative [36] suggests it is high under neo-classical aesthetics (that say: mirror the reality via certain rules of truth and beauty), and lower under romantic and nationalistic ideas (that say: a poem reflects a poet themselves, while also reflecting a little of a national spirit). This makes us expect a decrease in strength of semantic halo over time in European poetry. At the same time we don’t expect individualistic poetics to completely diffuse the halo effect: the structural difference in meters would continue to maintain distinction in the semantics.

Given the main assumptions, we argue that any poetic tradition that allows for structurally distinct poetic forms to exist over an extended time (whatever the case) should exhibit the semantic halo effect. This study, however, seeks to find the effect mainly in three closely related European accentual-syllabic (AS) traditions: Czech, German and Russian. In addition, we look for further evidence in English poetry and early modern Dutch songs. Since all traditions use the same versification system, metrical forms are inherently comparable, which makes cross-cultural study possible. Data come from corpora of different designs (S1 Appendix), and even include different textual domains (Dutch songs). The heterogeneity of sources ensures that our findings would not result from some artifact of corpus construction. We devise a language-independent methodology to represent each poem as a set of semantic features (latent topics inferred from all available texts within a corpus) and rely on unsupervised clustering to assess the strength of meter-meaning relationships.

The main question of this study is whether poems written in a particular meter also tend to employ similar topics **(H1)**. We formalize this as clustering and classification tasks. Do poems written in one meter tend to group into coherent semantic clusters? And can a meter be recognized based on knowledge of nothing but the semantic features of corresponding poems?

These queries, in turn, raise the issue of semantic diffusion and retention. Diffusion would happen because of possible change in aesthetics and conformity pressure. We expect clustering to be less efficient in the later phases of a tradition compared to its early stages when we use the same sets of meters and similar sampling strategies **(H2)**. This should be evident in all the corpora except for the Dutch songs: texts come from the early modern period and represent a tradition grounded in oral performance, which depends on the reproduction of existing forms and is presumably less prone to long-term innovations.

Finally, we expect that despite the diffusion of the semantic halo, poetic traditions retain a historical connection to the halo’s earlier states. If this is true, then it should be possible to recognize meters from earlier periods using models only exposed to later data and vice versa **(H3)**.

To test these assumptions, we use the abstracted semantic features of poems (represented as topic probabilities) to recognize their metrical organization (represented as the unambiguous labels of metrical types, see Table 1). The distribution of topics or co-occurring groups of words in each poem is inferred with the aid of a generative Latent Dirichlet Allocation (LDA) model [37] trained for each lexically simplified corpus. Metrical labeling of poems combines information about the general metrical scheme used (e.g. iamb) and the particular type of this scheme (e.g. pentameter). We understand metrical types to be the main verse forms reproduced within and across traditions; they each have a distinct historical lineage that may be reconstructed to a greater or lesser extent. To take one example, English iambic pentameter, as introduced in the 14th century, may be traced to 10-syllable French isosyllabic meter, which developed from the Italian 11-syllable form. On the other hand, the so-called “ballad” meter (Iamb 4–3, “common measure”) originated from contacts between local English accentual verse and Latin vagant songs [20, 38, 39]. Where possible, the poems in our corpora were individually annotated with a single unambiguous label related to their metrical type. (The limitations of this approach are discussed in S2 Appendix).

We assume that meter & meaning association is impossible to trace at the level of a single poem since the semantic halo is not a deterministic mechanism [16] that prescribes meanings to texts. Rather this mechanism is probabilistic and observable at the level of central tendencies (which might, in turn, depend on the social transmission factors and aesthetic conventions of a group or a tradition). Given these factors and the skewed distribution of the metrical forms used in a tradition (see S3 Fig), we rely on an approximation of each meter’s semantic features based on random equal-sized samples of poems from a set of the most frequently used forms in the language.

Our analysis confirms the presence of the semantic halo of meter in all observed European traditions. In all cases except for the Dutch example, we also observe the predicted historical decline in the strength of this association. When the sample size is large enough,the cross-period classification also exceeds the random baseline in all cases. There are, however, significant differences among the corpora that may reflect different levels of exposure to change in the poetic traditions.

## Results

### Meter and meaning (H1)

We approach the association between meters and topics with a clustering setup: we expect that independent samples of poems written in the same meter, as represented by vectors of topic probabilities, will tend to group together. We limit the data to meters that are sufficiently common (# of poems > 500) and then divide them into random samples of 100 poems per meter and aggregate topic probabilities within each sample. *K*-means clustering is then used to group the samples into *k* clusters, with *k* being set as the number of distinct meters. The resulting clusters are compared against the true groups based on the metrical types using Adjusted Rand Index. While Rand Index measures purely the similarity between two cluster groups on the scale from zero (no agreement between the clusters) to one (clusters are the same), Adjusted Rand Index is the corrected-for-chance version of this score where 0 indicates a situation when clustering is no different from a random assignment of data to clusters and 1 indicates perfect match between the two groups [40].

**Fig 1:**
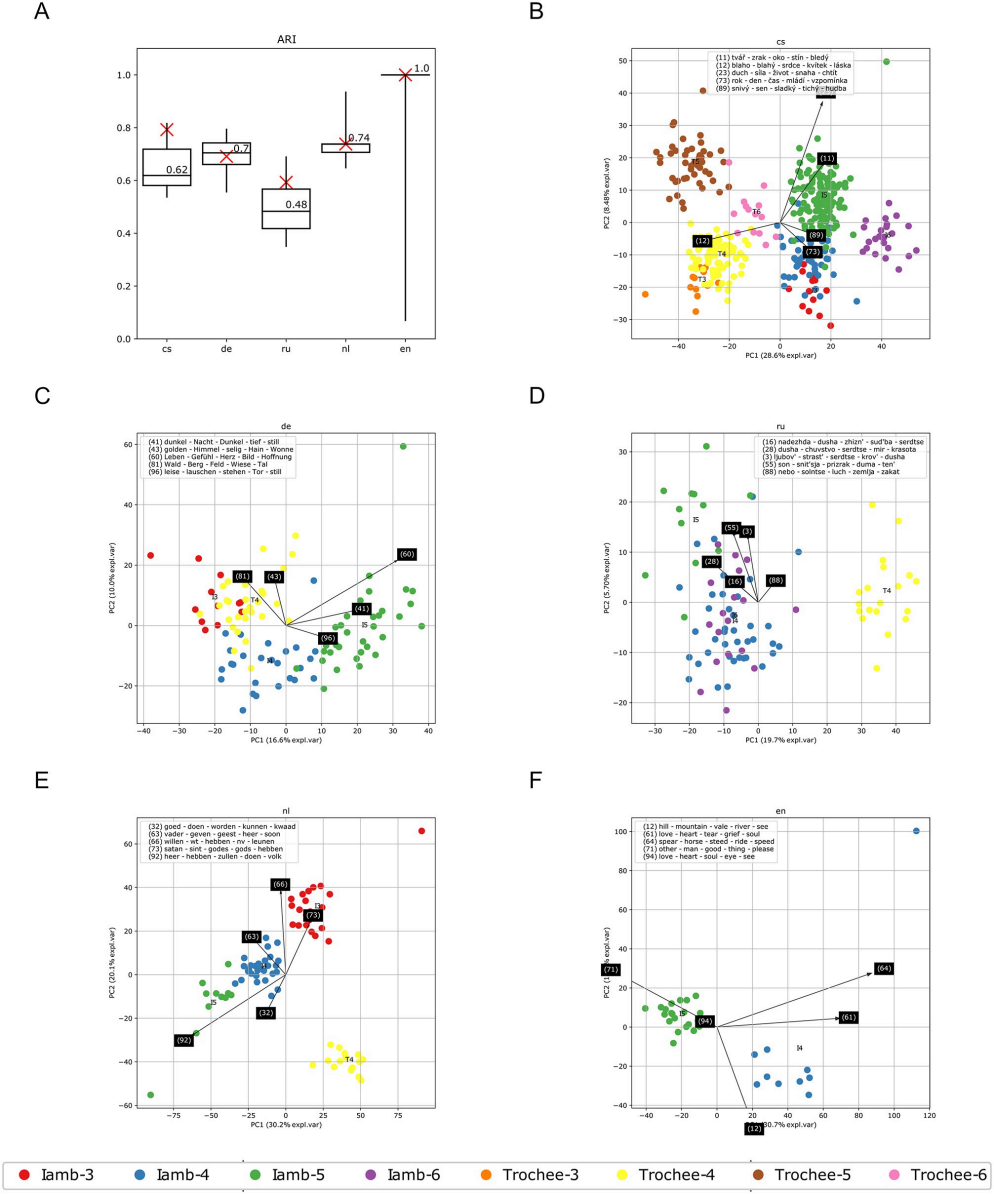
Random 100-poem samples taken without replacement per meter in vector spaces defined by LDA topic models. (A) Adjusted Rand Index of *k*-means clustering (whiskers give the 5th- to 95th-percentile range). 10,000 random samplings. Crosses show the ARI of the samplings presented in PCA biplots. PCA biplots of (B) Czech (8 meters), (C) German (4 meters), (D) Russian (4 meters), (E) Dutch (4 meters) and (F) English data (2 meters) respectively with eigenvectors for the 5 most contributing topics. Single random sampling.

The procedure is repeated 10,000 times with each corpus (Fig 1A). All results point to a high level of association between metrical forms and the semantic features of corresponding poems in each of the observed corpora. This provides strong support for the presence of the semantic halo across all traditions and confirms H1. To illustrate the extent of the association between form and meaning, one such random sample from each corpus is represented by means of two-dimensional PCA biplots (Fig 1B–1F).

**Fig 2:**
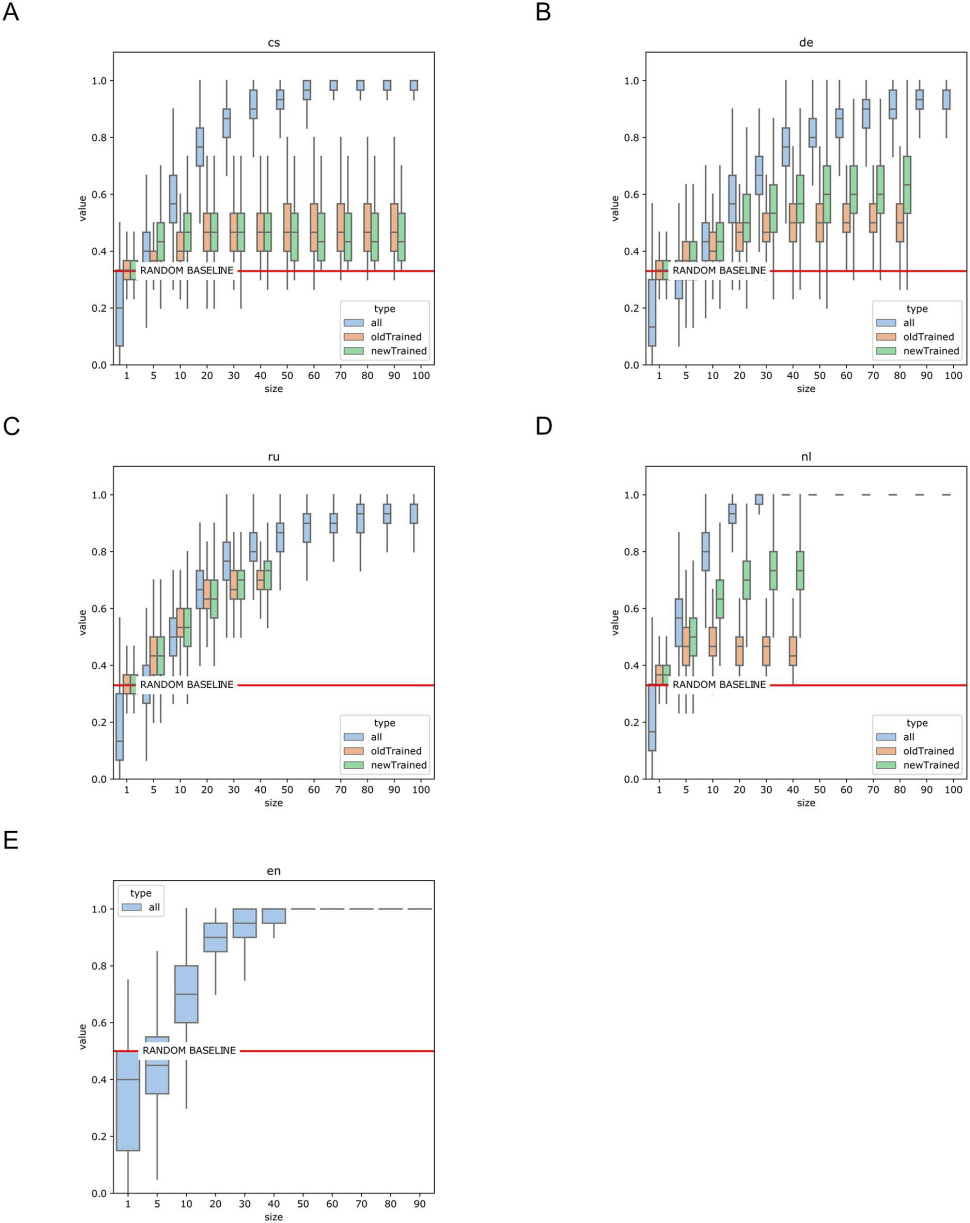
Accuracy of SVM classifications. Predicting meter with vectors defined by topic probabilities in random samples of poems (sample size ∈{1, 5}∪{10, 20, …, 100}). (1) trained and tested on the entire dataset (leave-one-out cross-validation), (2) trained on earlier data and tested on later data, (3) trained on later data and tested on earlier data. 10,000 iterations. (A) Czech, (B) German, (C) Russian, (D) Dutch, (E) English.

An additional supervised classification run corroborates the evidence with even smaller samples (Fig 2: first boxplot series).

### Diffusion over time (H2)

We expect the semantics of meter to become less recognizable over time: poems emerged within aesthetics that reportedly shifted from normative to individualistic [36] which would allow to untie meters from strict genre expectations. This hypothesis is, however, generally limited to canonical 19th-century poetry, and there is no reason to apply it to early modern popular songs (i.e. our Dutch corpus). As a tradition which relied on oral performance and the persistence of popular melodies, these songs are expected to maintain more stable generic lineages.

To test the hypothesis, we split each corpus into two subcorpora based on the date of publication of particular poems. (Both the amount of data at our disposal and the distribution of meters over time prevent us from dividing the corpora according to a more granular chronology or partitioning the English data in a similar way.) In each subcorpus, we test the strength of the association between metrical labels and sample-wide aggregated semantic features in the manner described above. To ensure comparability, we use the same set of meters and draw a fixed number of samples from the two parts of each corpus.

Based on H2, we expect to see a decrease in clustering accuracy in the later stage of the timeline when compared to the earlier part. We observe this trend in the Czech, German and Russian corpora but not in the the Dutch songs where forms and their semantics maintain similar levels of association over 200 years (Table 2). To formally address the trend in three main languages, we fit a Bayesian regression model on a subset of our clustering results allowing interaction between period and language groups. We directly estimate the difference in clustering (S6 Fig) between periods across languages using predictive draws from posterior (Czech: *β* = −0.08, lower 95% CI = −0.1, upper = −0.06; German: *β* = −0.48, lower CI = −0.52, upper = −0.44; Russian: *β* = −0.11, lower CI −0.15, upper = −0.06).

**Table 2:** Adjusted Rand Index of *k*-means clustering in different periods (random 100-poem samples). 10,000 iterations.

Language	Time span	ARI		Meters	# of samples per meter
		mean	std. dev.		
Czech	1800–1859 1860–1919	0.994 0.878	0.034 0.152	I5, T4, T5	5
German	1750–1824 1825–1899	0.958 0.444	0.098 0.226	I4, I5, T4	5
Russian	1800–1859 1860–1919	0.715 0.642	0.181 0.218	I4, I5, T4	5
Dutch	1550–1649 1650–1749	1 0.995	0 0.013	I3, I5, T4	4

### Retention of forms over time (H3)

Despite changes over time in the strength of the semantic halo, we expect to see historical continuity in the use of various forms so that we may predict the later stages of a meter’s semantics by knowing its earlier semantic features and vice versa. To test this, we perform supervised classification on each corpus: the training set is restricted to samples from one subcorpus while the test set only includes poems from the other one. The results show high variation (Fig 2) across the corpora, which may reflect differences in their sources and/or historical idiosyncrasies in the usage of meter. The Russian tradition exhibits the most stable use of the main metrical forms over the observed period; there is little difference between past and future forms. In contrast, both the German and Dutch corpora show higher semantic recognizability from the future to the past than in the opposite direction. The assymetry suggests semantic accumulation over time, a pattern where later metrical semantics “enclose” [41, 42] the early usage of a form but are already too different to be recognizable from the past. The recognizability of Czech forms stays stable at a level barely above the random baseline: it is likely connected to this tradition being in a volatile establishment state and changing its metrical preferences midway through the 19th century (S4 Fig).

Overall these results provide weak support for H3, but also highlight the variation in the histories of meter usage.

## Discussion

Our findings strongly support the association between poetic meter and meaning, providing evidence for the theory of the semantic halo. At least within certain accentual-syllabic systems, European poetic traditions demonstrate their use of conservative mechanisms to produce and retain meaning. Metrical forms attract arbitrary semantic features that are reproduced when the form itself is reproduced. The precise mechanisms of this attachment are yet to be understood but different dynamics may be at play. These could include forces intrinsic to the texts (e.g. the mnemonic function of a meter [34]), institutional factors (popularity, example-based teaching, the literary canon [43]) and transmission biases, like conformity and prestige.

By focusing on semantic generalization over poetic nuance, our approach is able to demonstrate the overarching tendencies that distinguish meters from one another. As Fig 2 and distinctive topics for each meter suggest, the most striking thematic distinction occurs between trochaic and iambic meters. Globally, the trochee is tied to a song genre and is associated with the themes of love, vernacular language and national romantic sentiment. This highlights the trochee’s historical roots: across Europe, folk songs were associated with the trochee or trochaic rhythm [16] and they often took form of regular trochaic meters in modern versification systems. Iambic forms, on the other hand, can be distinguished by topics that point to a learned poetic style: introspective reflections, religious and existential themes. Dutch songs demonstrate the difference most clearly: in Dutch, iambic songs cover religious topics and are the products of an educated, written tradition, while trochaic songs maintain vernacular themes (love, joy, work) that tie them directly to their origins in oral culture.

The semantic tradition that separates iambs from trochees should not, however, be seen as universal. The semantic similarity of trochee-4 and iamb-3 in German, for example, points to the similar usage of these two meters and their historical origins in the European tradition of “anacreontics” [16]: lyrical songs of feasts, wine and love that are associated with the neo-classical tradition. During the Romantic period, anacreontics easily mixed with rediscovered folk songs and vernacular language, which made iamb-3 gravitate towards the trochaic meaning space. The semantic proximity of these two forms is also evident in Russian verse [25], which partially inherited the use of this meter from German. Specific cases with a well-documented history are also captured by the model. For example, trochee-5 in Czech was the meter embraced by elites during the national revival and its distinctive topics reflect national pathos, folk imagery and civil uprising [44]. Russian trochee-5, on the other hand, was initially a rare form. A single extremely popular poem in this meter launched a tradition that bears some similarity to the “founder” poem to this day: the LDA model is able to recover topics that refer to its semantic halo that scholars summarise as “introspective travel on the road (real or metaphorical) at night” [16].

Our conclusions about similar meter and meaning association processes in European verse have obvious limitations. First, the three main traditions observed in our study are closely related and did not develop in isolation: German metrical models played a foundational role in establishing modern verse in both Russian and Czech. This means that deeply rooted metrical associations (e.g. the difference between a trochee and a iamb) may result from common origins or cultural proximity, and not from differentiation as a common function of meter. Second, in this work we aggregate different stanzas and rhyme schemes under a few metrical labels, but these forms often had their own distinct usage traditions. In fact, stanza organization may be more relevant than meter for distinguishing genres in languages where verse regularity is based exclusively on the number of syllables (e.g. French, Italian, Spanish). In other words, we cannot define a “poetic form” universally for every poetic tradition, and broader comparative research will need to operate at several levels of abstraction.

We are hopeful that our findings will advance the conversation in metrical studies since they provide the first large-scale formal evidence of an association between meter and meaning in Western poetry. They also pave the way for the incorporation of the explicit modeling of historical processes into literary studies: mechanisms that drive the formation and change of the semantic halo generally remain a mystery and would require well-defined models that link individual interactions to the observed patterns. This turn to cultural evolutionary framework and cultural transmission models could establish common ground with linguistics, anthropology and social sciences for understanding factors behind the changes and continuities in cultural traditions. Identifying the mechanisms that limit cultural transmission [45] and generate long-term patterns in creative domains is also important if we are to understand the rate of cultural evolution [46, 47] and the shape of cultural phylogenies [48–51]. If trochaic tetrameters are more semantically similar to their trochaic tetrameter “ancestors” than to any other meter, the form alone could be responsible for establishing and maintaining the divergent traditions within a creative domain or, as we usually call them, genres.

## Materials and methods

### Corpora

Our research uses five metrically annotated poetry collections in normalized orthography, each of which concerns one language tradition: Czech [52, 53], Dutch [54], English [55], German [56, 57] and Russian [58, 59]. These collections have disparate sources and vary in size, chronological scope, general composition principles and survivorship bias (the Russian corpus, for example, favors poems that were reprinted in 20th-century scholarly editions). A summary of the corpora before filtering and pre-processing is provided in Table 3. For more details about each collection and a summary statistics, see S1–S4 Figs.

**Table 3:** Summary of poetry corpora used.

Language	Texts	Period	Tokens
Czech	69,760	18–20th c.	13,100,898
German	53,608	16–20th c.	10,462,211
Russian	17,900	18–19th c.	3,329,352
Dutch	22,297	1550–1750	6,562,888
English	6,448	16–19th c.	2,126,436

We rely on poetic meters to distinguish between verse forms. Meters—the idealized rules of a text’s prosodic composition—arise from the stable recurring rhythmic patterns that organize language prosody in particular ways. In accentual-syllabic (AS) verse, meters are organized around the distinction between weak and strong stress. Regularity in AS systems is based on the recurring groups of syllables: a two-syllable unit in a weak-strong sequence is called a iambic foot; a sequence of feet organizes a poetic line (e.g. iambic pentameter). As a group of contextually aligned lines, a poem gives us information about the metrical type it follows. In classical AS systems, homogeneous metrical types were dominant in poems (e.g. all lines followed the general pattern of iambic pentameter) although the use of alternating types and free form is not uncommon. In our research, we focus on iambic and trochaic metrical types—the most widespread ways to organize verse in European AS traditions.

### Meter recognition

All the collections in our study were metrically pre-annotated on a line-by-line basis using language-specific rule-based algorithms [52, 57, 58, 60] or manual methods [54]. Since in all five traditions the dominant versification system is accentual-syllabic, all prosodic patterns and verse organization principles are comparable. However, the task of formally describing a poem in a collection is not straightforward since “metrical form” may be interpreted on several levels (see S2 Appendix).

Where possible, each poem in a corpus was assigned a single unambiguous metrical label (e.g. “iamb-4” (I4), “trochee-5” (T5), etc.). This process involved some heuristics to determine labels: in line with existing annotation simplification principles [16], we considered a poem to be of a particular metrical type if at least 80% of its lines conformed to that same pattern. All heterogeneous cases or non-metrical poems were left unmarked. (Our models were built on all of the available texts, but only the labeled ones were used in the analysis). The loss of data at different filtering steps is presented in S4 Table. Trochee and iamb cover the majority of recognized pool of metrical poems.

### Pre-processing

All corpora were initially filtered by size to exclude poems that were too short or too long so that the document sizes would remain comparable in the LDA model. We also excluded early modern poems from German and English (S2 Fig) to maintain a comparable chronological range across the corpora. Each corpus went through lemmatization and part-of-speech tagging (MorphoDiTa [61] was used for the Czech corpus while TreeTagger [62] was employed for all the other corpora). Afterwards, we applied lexical simplification to words outside the 1000-most-frequent list so that the frequency distribution was less sparse (LDA models are susceptible to noise and work better with a reduced vocabulary [63–65]). Low frequency words were replaced with one of their more common contextual neighbors if that neighbor appeared in the list of the 10 semantically closest words. Semantic similarities were determined independently for each corpus with word-embedding models that had been trained on the respective collections to capture the specific semantic relationships of poetic language. For the word-embedding models, we used word2vec [66] implementation from the Python gensim framework [67].

### Semantic features

Our study of the overarching semantic relationships of metrical forms required some abstract representation of poetic language that would allow us to summarize a poem’s “content” at a general level. We used LDA topic models [37] trained for each of the collections on the non-aggregated data. An LDA model infers topics—groups of co-occurring words—from a collection without supervision. Since LDA is a generative algorithm, each poem in a corpus can be uniformly represented by the topic probabilities that generate its distribution of words. In cultural analytics, topic modeling has become a common way of inferring higher-order semantic properties from a collection of texts so that they can be used for further analysis and reasoning [11, 46, 68, 69]. While it is less efficient when used with shorter texts [70], it has proven to be generally applicable to poetry without any major reported drawbacks [25, 71, 72].

Topic modeling, lexical simplification and lemmatization served another important goal in our study: they mitigated the effects of metrical patterns on morphology and sentence structure [5, 35]. We additionally checked that our results would not be fully explained by pure morphology-based clustering (S2 Table).

Our results are reported based on an LDA model trained with 100 topics, but our findings remain robust with the change in the number of topics (we tested models with 20, 50, 100 and 150 topics, S1 Table). The choice of model is therefore not particularly important for the study design. On the other hand, increasing the number of topics may increase the human interpretability of the relationships.

### Clustering and classification

Most of the analysis in this study relies on unsupervised approach to classification. There were two reasons for this: the first was data scarcity because of uneven popularity of different meters. The second reason was the risk of over-fitting: in testing associations between meter and meaning, we wished to capture naturally emerging relationships rather than imposing a fixed view about known “classes” on the data.

We used supervised classification solely to model chronological perspectives on meter recognizability (H3). In this case, the training of the model was stratified by time so that learned classes only incorporated “past” or “future” knowledge about the meter usage in a tradition.

For *k*-means clustering and the Adjusted Rand Index [40] calculation, we used the implementation provided by the Python library scikit-learn [73] (sklearn.cluster.KMeans). The number of clusters was set as the number of distinct meters found in the dataset.

To test H3, Support Vector Machine classification was also performed with the scikit-learn library, (sklearn.SVM.SVC). We used the classifier with a degree-3 polynomial kernel.

### Regression

To model difference between periods across languages we fit a Bayesian regression using brms interface in R. We use a random subset of 100 clustering results per language per period to introduce some uncertainty to the model. We predict clustering results (ARI) with two-way interaction between periods and languages. Since response ARI values cannot go over 1 and also exhibit extreme uniformity in some cases (early Czech and early German), we assume a truncated normal distribution for ARI with the upper bound on 1. To ensure convergence, we subtract a small dummy value (0.02) from ARI measures to push it slightly away from the truncation boundary (ARI can’t go over 1, but in some cases takes values below 0). We use weakly informative priors on “slope” parameters (*β* ∼ *Normal*(0, 0.1)). See coefficients (S5 Table) and predictive posterior distribution (S6 Fig) of ARI values across languages.

## Supporting information

S1 Appendix Corpora details. (PDF)

S2 Appendix Metrical annotation. (PDF)

S1 Fig Distribution of poem sizes. Red vertical lines mark our filtering cutoffs.
(TIFF)

S2 Fig Chronological distribution of poems. (TIFF)

S3 Fig Distribution of metrical types per tradition. (TIFF)

S4 Fig Historical changes in the use of meters and proportion of given forms in the corpora. In the Czech works, we see a radical shift from trochee- to iamb-based works. There is also a gradual rise in trochee usage in the Dutch song corpus. This likely reflects the increasing representation folklore texts in print.
(TIFF)

S5 Fig PCA biplots of (A) Czech and (B) German variants of iambic pentameter with eigenvectors of the five most influential topics. Single random sampling. Each variant is defined as the shortest re-occurring pattern of line endings within the poem (i.e. whether these are masculine, feminine or dactylic).
(TIFF)

S6 Fig Posterior predictive distribution of ARI across languages and periods. Colored points show posterior means, error bars show 95% credible intervals. Grey points represent empirical values (jitter added to x-axis for better readability).
(TIFF)

S1 Table Random 100-poem samples taken without replacement for each meter in vector spaces defined by LDA topic models(20, 50, 100, 150 topics). The results of our H1-related analysis show no qualitative variation regardless of the number of topics used to train an LDA model. We repeated the analysis in its full form (10,000 clustering iterations) for four different LDA models and report the Adjusted Rand Index mean along with the interquartile range.
(PDF)

S2 Table Random 100-poem samples taken without replacement for each meter in vector spaces defined by part-of-speech frequencies. Adjusted Rand Index of *k*-means clustering. Accentual-syllabic meters are systems of limitations superimposed on language. As such, they transform natural morphological and syntactical affordances [35]. This is why we can distinguish poetry from prose so easily based on word frequencies [5]. In addition, the distribution of parts of speech differs across metrical forms. Some words are more common in ternary meters simply because they are less likely to appear in binary meters for prosodic reasons. This has little connection with the semantics of meter but instead reflects the structural properties of verse. To make sure our pre-processing steps mitigated the problem of morphological differences, we repeat the clustering procedure from the H1 analysis. Here we use the frequencies of parts of speech that were included in LDA model (nouns, adjectives, verbs) as a feature set. S2 Table. shows that accuracy is visibly lower in this case than when clustering is topic-based.
(PDF)

S3 Table Distinctive topics for the most common meters. The most distinctive topics for the most common meters. For each meter, topic probabilities are averaged across the entire corpus. These values are transformed into *z*-scores across particular meters. The table shows the five topics with the highest *z*-scores for each meter.
(PDF)

S4 Table Corpora filtering. Step 1 gives the number of poems after exclusion of other than accentual-syllabic poems (these include for instance free verse, accentual verse, syllabic verse), poems where less than 80% of lines are written in a single meter, poems outside the selected time span and poems outside the required length span (4 to 100 lines). The figure in parentheses gives how many poems remain after this step as compared to the entire corpus. Step 2 gives the number of poems when keeping only the most common iambic and trochaic meters. The figure in parentheses gives how many poems remain after this step as compared to the previous one.
(PDF)

S5 TableH2 model coefficients, truncated normal.Fit of a Bayesian regression model with Hamiltonian Monte Carlo sampling (4 chains, 2000 iterations, 1000 warmup); brms formula: *ARI*|*trunc*(*up* = 1) ∼ 1 + *period* * *language*. Categories are index-coded, reference level for predictors is Czech for languages and early for period. Bulk_ESS and Tail_ESS are measures of effective sample size. at 1 signifies chain convergence. effective sample size. $\hat{R}$ at 1 signifies chain convergence.(PDF)
