# Supplementary material for: Semantics of European poetry is shaped by conservative forces: The relationship between poetic meter and meaning in accentual-syllabic verse
Source: PLoS One. 2022 Apr 12;17(4):e0266556. doi: 10.1371/journal.pone.0266556 (PMC9004753; doi:10.1371/journal.pone.0266556)
Supplement: S1 Appendix — (PDF) [file pone.0266556.s001.pdf]

# S1 Appendix. Corpora details

- The Czech data come from the Corpus of Czech Verse created by Petr Plecháč and Robert Kolár. [1,2] The entire dataset is available at https://github.com/versotym/corpusCzechVerse.
- The German data come from the Metricalizer corpus created by Klemens Bobenhausen and Benjamin Hammerich (https://metricalizer.de/) [3,4]. This dataset is proprietary and was kindly provided by its creators for this study.
- The Russian corpus is part of the Russian National Corpus which is available to researchers upon request [5,6]. The corpus is metrically pre-annotated but our study uses an improved rhythm recognition algorithm developed by Yurii Zelenkov [7].
- English texts come from the Gutenberg English Poetry Corpus compiled by Arthur M. Jacobs. [8] Metrical annotation was performed using the Python package `Prosodic` developed by Ryan Heuser (https://github.com/quadrismegistus/prosodic/).
- The early modern Dutch songs used in this study are part of the Dutch Song Database (www.liederenbank.nl) compiled and hosted by the Meertens Institute in Amsterdam. The database contains more than 175,000 songs in Dutch or Flemish that date from the Middle Ages through to the twentieth century. The genres include love songs, satirical and religious works and children's songs. The main sources are songbooks, song sheets (broadsides), song manuscripts and field recordings.

  The Czech, German and Russian corpora are the main focus of this study: the poems in these collections cover a comparable cultural niche (prestige poetry) and time span. The Dutch texts come from early modern printed song collections of various sources and, thus, reflect a specific strand of poetic textual production and circulation. The English collection, on the other hand, contains works scattered over a significant time frame; particular periods are represented by few texts, and there is little metrical variation. As a result, we use the English & Dutch collections only as secondary sources. They show the general validity of our claims for material with substantially different structures and origins.

## References


1. Plecháč P, Kolár R. The Corpus of Czech Verse. Studia Metrica et Poetica. 2015;2(1):107 – 118. doi:10.12697/smp.2015.2.1.05.
2. Plecháč P. Czech Verse Processing System KVĚTA – Phonetic and Metrical Components. Glottotheory. 2016;7(2):159 – 174. doi:10.1515/glot-2016-0013.
3. Bobenhausen K. The Metricalizer: Automated Metrical Markup for German Poetry. In: Küper C, editor. Current Trends in Metrical Analysis. Frankfurt am Main: Peter Lang; 2011. p. 119–131.
4. Bobenhausen K, Hammerich B. Métrique littéraire, métrique linguistique et métrique algorithmique de l'allemand mises en jeu dans le programme Metricalizer2. Langages. 2015;199:67–87. doi:10.3917/lang.199.0067.

5. Grishina E, Korchagin K, Plungian V, Sichinava D. Poeticheskii korpus v ramkah NKRIA: obschaia struktura i perspektivy ispolzovania. In: Natsionalnii korpus russkogo iazyka: 2006-2008. Novye rezultaty i perspektivy. St. Petersburg: Nestor-Istoria; 2009. p. 71–113.

6. Korchagin K. Poezija XX veka v poeticheskom podkorpuse Natsional'nogo korpusa russkogo iazyka: problema reprezentativnosti. Trudy instituta im VV Vinogradova. 2015;6:235–256.

7. Šeļa A, Plecháč P. The case of (pseudo-)Batenkov: Towards a formal proof of literary forgery. In: Plecháč P, editor. Versification and Authorship Attribution. Prague: Karolinum/ICL; forthcoming. p. 80–91.

8. Jacobs AM. The Gutenberg English Poetry Corpus: Exemplary Quantitative Narrative Analyses. Frontiers in Digital Humanities. 2018;5:5. doi:10.3389/fdigh.2018.00005.
