# Supplementary material for: Semantics of European poetry is shaped by conservative forces: The relationship between poetic meter and meaning in accentual-syllabic verse
Source: PLoS One. 2022 Apr 12;17(4):e0266556. doi: 10.1371/journal.pone.0266556 (PMC9004753; doi:10.1371/journal.pone.0266556)
Supplement: S2 Appendix — (PDF) [file pone.0266556.s002.pdf]

# S2 Appendix. Metrical annotation

A poem's form may be recognized at different levels of granularity. Consider *The Raven* by Edgar Allan Poe:

1. Generally, the metrical pattern of this poem is based on the trochee, i.e. it is made up of a recurring binary rhythmic unit (foot) in which a strong (stressed) syllable is followed by a weak (unstressed) one.

2. Most lines in *The Raven* consist of eight trochaic feet: the overall meter is defined as trochaic octameter (shortened here to "Trochee-8").

3. Readers of *The Raven* will notice that its octametric lines are organized into stable five-line units followed by a last (sixth) line that is shortened to tetrameter. At a stanzaic level, this form could be coded as Trochee-888884. Clearly it is so specific that any recurrence of it in other works signals a connection to Poe's poem.

4. This formal description could be expanded to include rhyme patterns. For the final rhymes in *The Raven* , the coding would be: Trochee-888884-ABCBBB. Research suggests that in some cases (e.g. cultural borrowings from the syllabic versification to the accentual-syllabic traditions), distinctive rhyme schemes may be associated with semantic traditions independently of meter. [1].

5. Finally, the rhythm of poems is sensitive to the pattern of line endings, i.e. whether a rhyme ends on a stressed syllable (an acatalectic or masculine rhyme) or an unstressed one (a catalectic or feminine rhyme). When this factor is taken into account, the coding of *The Raven* would look like Trochee-888884-ABCBBB-fmfmmm.

As this summary shows, form may be seen as hierarchically organized: variations may occur from the level of the most abstract pattern (the trochee) through to the specific implementation of a meter in a highly regularized stanza. In this study, we aim for the mid-level of this hierarchy (number 2 on the list above). We apply a metrical type that is specific enough to register as structurally different but also abstract enough to be reasonably represented across the corpora and resistant to annotation errors and inconsistencies. This means that our analysis focuses on general relationships among meters but is not sensitive to possible variations within a particular form. Clearly the semantic representation of this general metrical type will be biased towards its most frequent metrical arrangements. Figure S5 shows that the most common metrical variants within a metrical type (e.g. different variants of Iamb-5) also remain semantically recognizable.

## References


1. Polilova V. Spanish Romancero in Russian and the semantization of verse form. Studia Metrica et Poetica. 2018;5(2):77–108. doi:10.12697/smp.2018.5.2.04.
