# Supplementary figures and images for: Semantics of European poetry is shaped by conservative forces: The relationship between poetic meter and meaning in accentual-syllabic verse
Source: PLoS One. 2022 Apr 12;17(4):e0266556. doi: 10.1371/journal.pone.0266556 (PMC9004753; doi:10.1371/journal.pone.0266556)

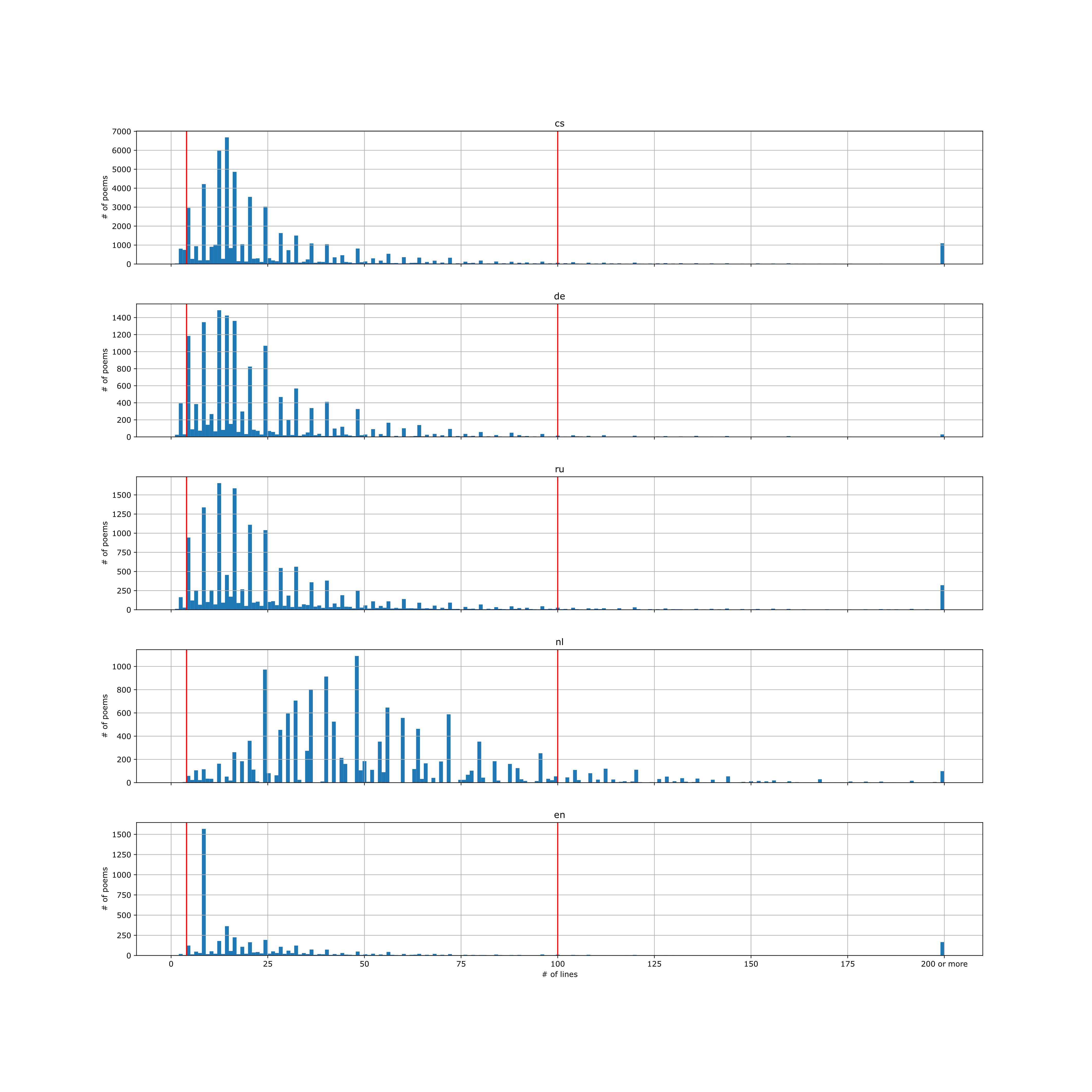

Supplement: S1 Fig — Red vertical lines mark our filtering cutoffs. (TIFF) [file pone.0266556.s003.tiff]

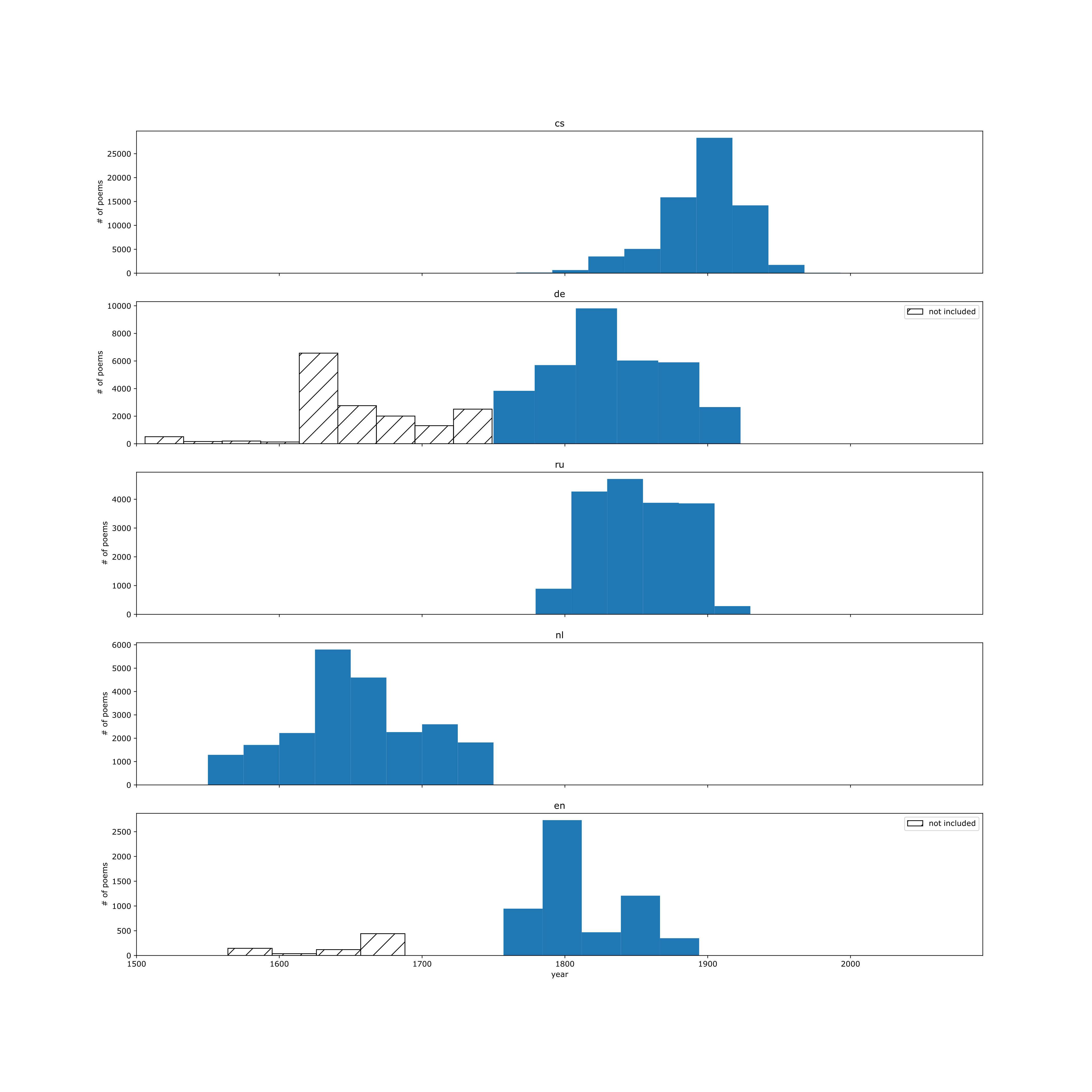

Supplement: S2 Fig — (TIFF) [file pone.0266556.s004.tiff]

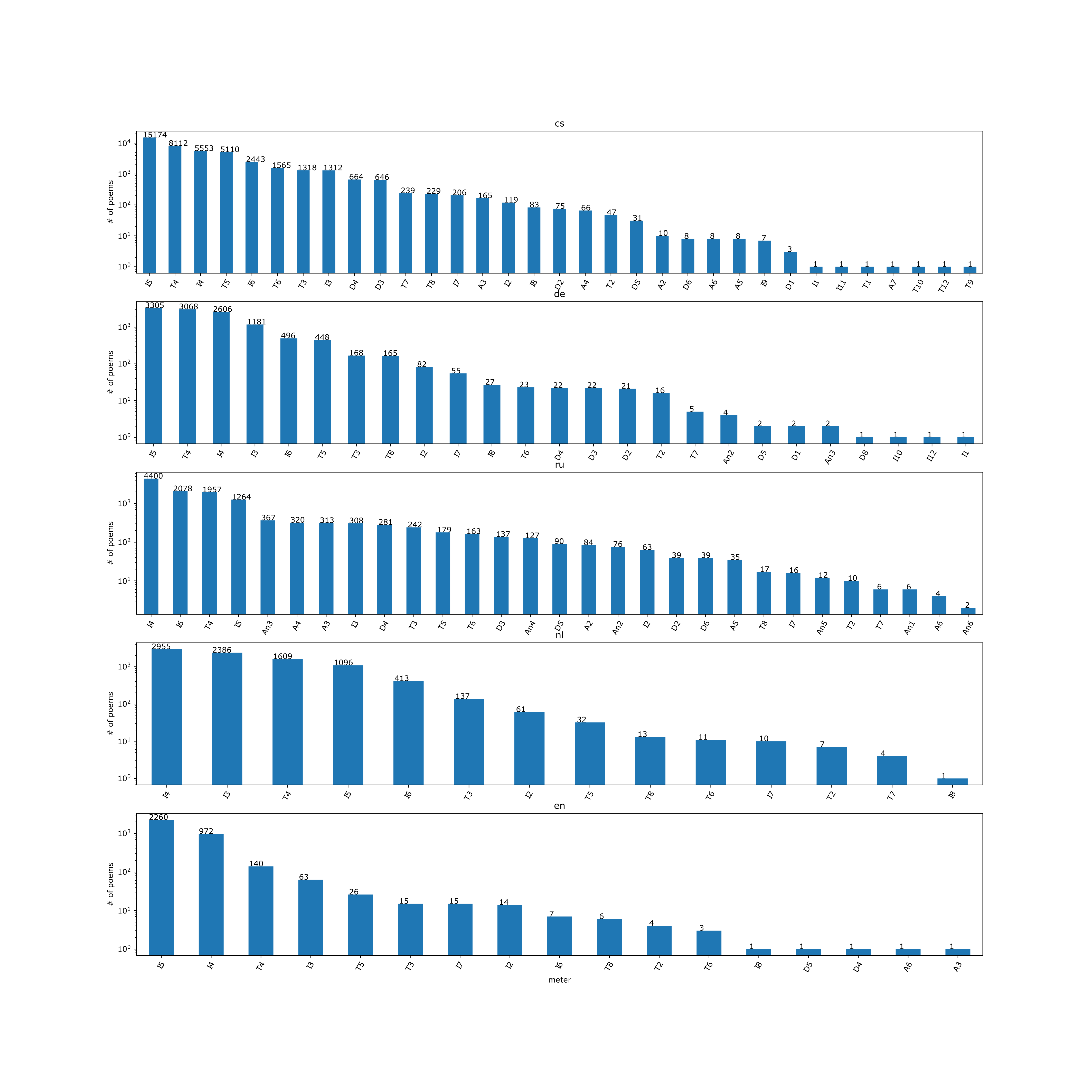

Supplement: S3 Fig — (TIFF) [file pone.0266556.s005.tiff]

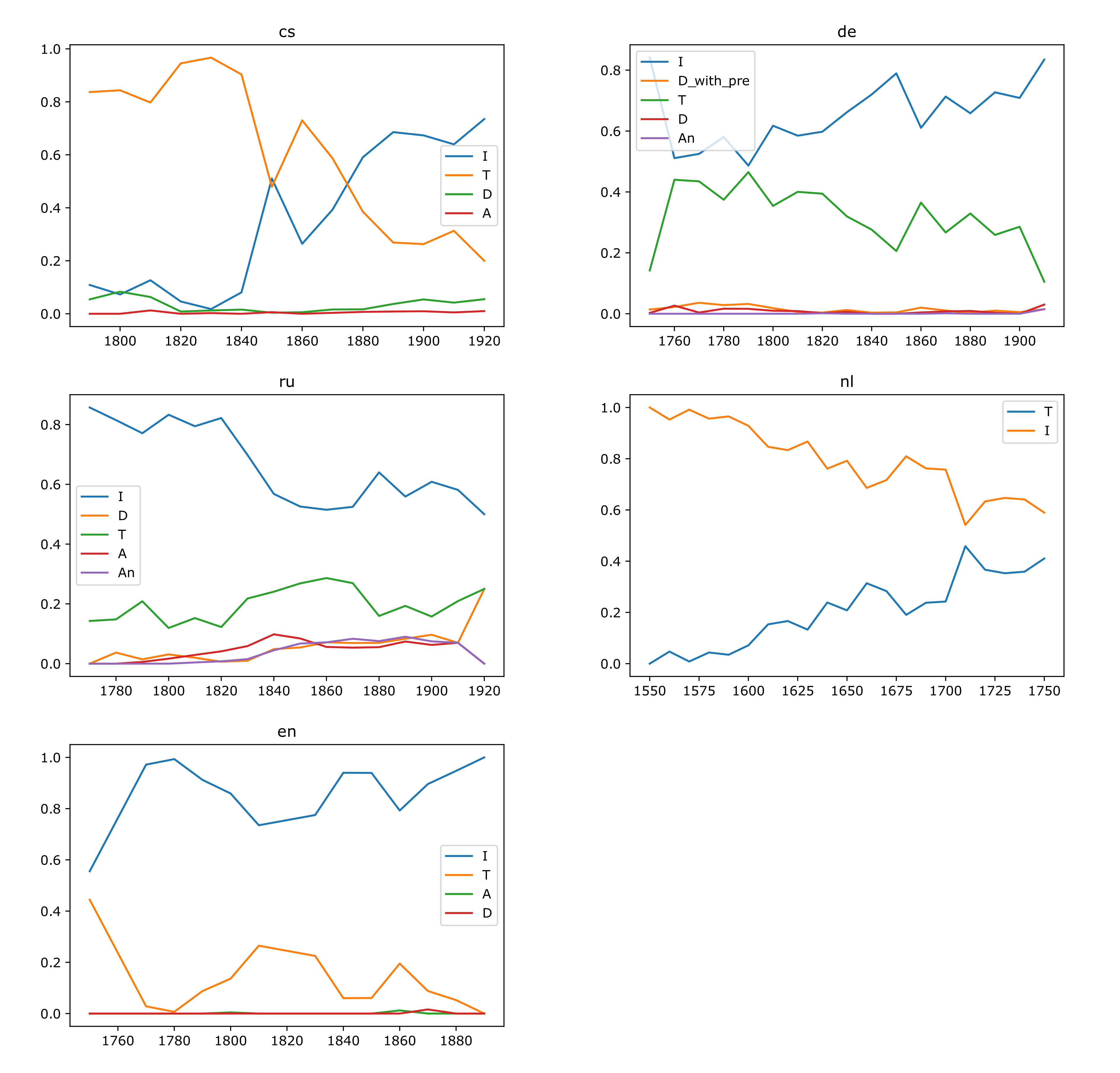

Supplement: S4 Fig — In the Czech works, we see a radical shift from trochee- to iamb-based works. There is also a gradual rise in trochee usage in the Dutch song corpus. This likely reflects the increasing representation folklore texts in print. (TIFF) [file pone.0266556.s006.tiff]

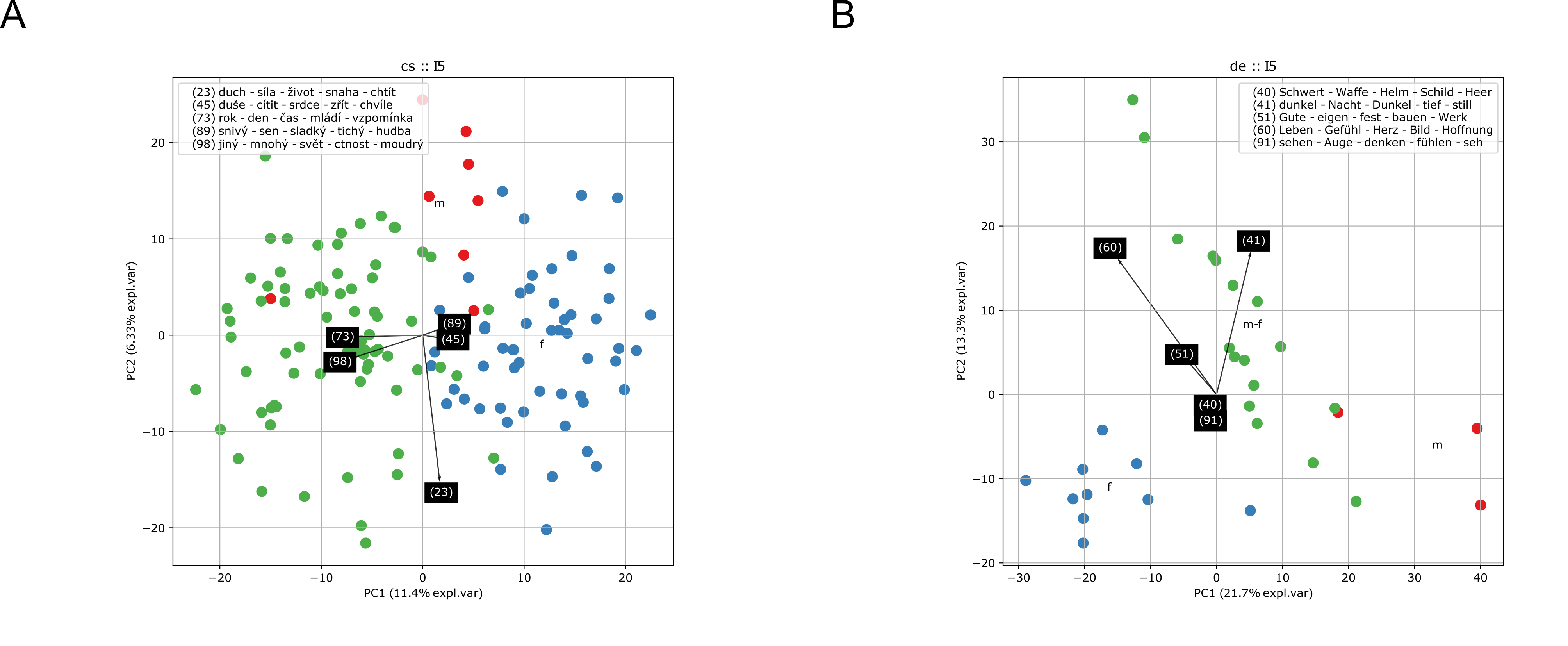

Supplement: S5 Fig — Single random sampling. Each variant is defined as the shortest re-occurring pattern of line endings within the poem (i.e. whether these are masculine, feminine or dactylic). (TIFF) [file pone.0266556.s007.tiff]

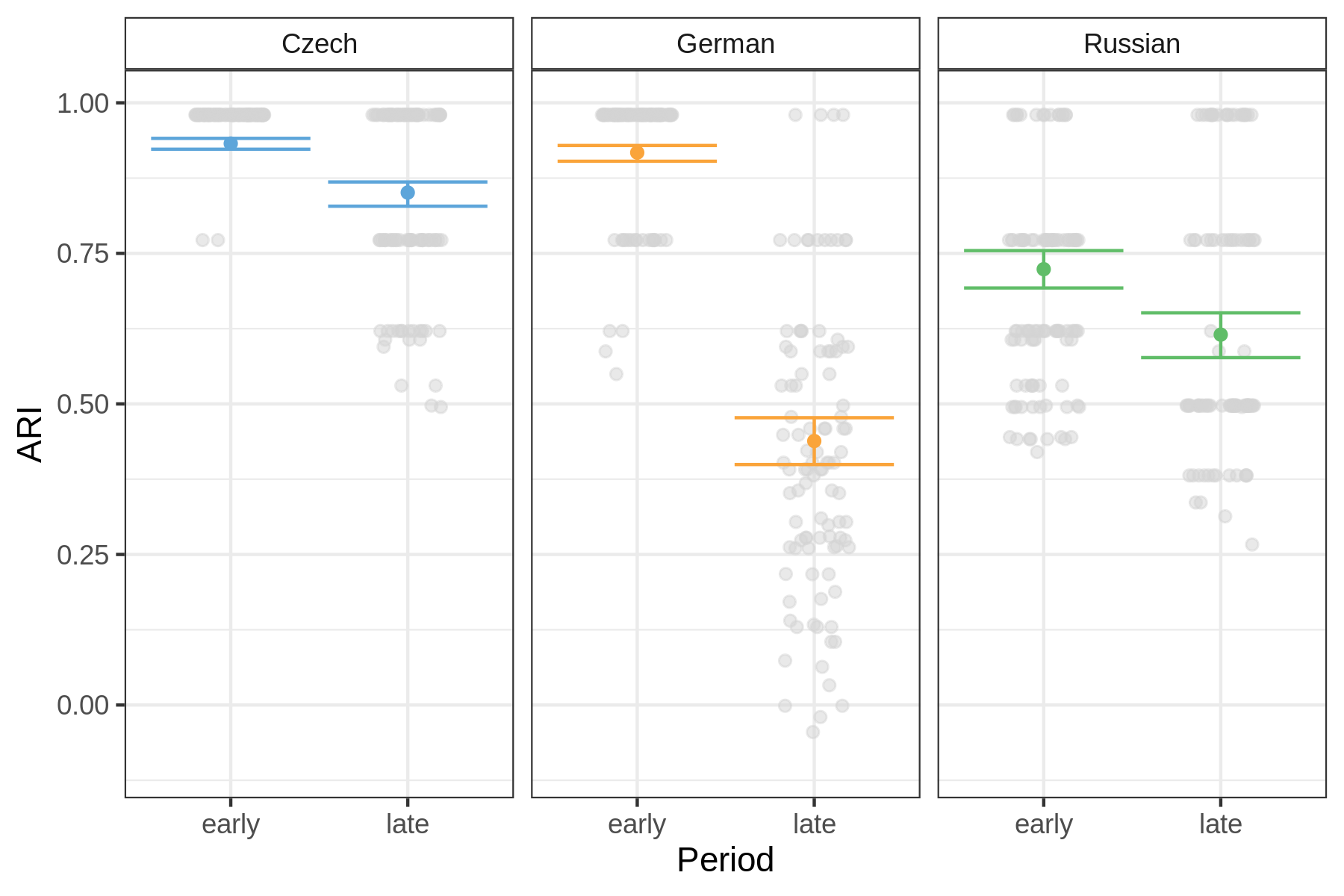

Supplement: S6 Fig — Colored points show posterior means, error bars show 95% credible intervals. Grey points represent empirical values (jitter added to x-axis for better readability). (TIFF) [file pone.0266556.s008.tiff]
