# Supplementary material for: Semantics of European poetry is shaped by conservative forces: The relationship between poetic meter and meaning in accentual-syllabic verse
Source: PLoS One. 2022 Apr 12;17(4):e0266556. doi: 10.1371/journal.pone.0266556 (PMC9004753; doi:10.1371/journal.pone.0266556)
Supplement: S1 Table — The results of our H1-related analysis show no qualitative variation regardless of the number of topics used to train an LDA model. We repeated the analysis in its full form (10,000 clustering iterations) for four different LDA models and report the Adjusted Rand Index mean along with the interquartile range. (PDF) [file pone.0266556.s009.pdf]

	Czech		German		Russian		Dutch		English	
# of topics	median	IQR	median	IQR	median	IQR	median	IQR	median	IQR
20	0.57	0.05	0.72	0.12	0.39	0.05	0.92	0.23	1	0
50	0.64	0.19	0.74	0.07	0.45	0.12	0.74	0.03	1	0
100	0.62	0.14	0.7	0.08	0.48	0.15	0.74	0.03	1	0
150	0.64	0.2	0.71	0.08	0.4	0.11	0.71	0.06	1	0
