# Supplementary material for: Semantics of European poetry is shaped by conservative forces: The relationship between poetic meter and meaning in accentual-syllabic verse
Source: PLoS One. 2022 Apr 12;17(4):e0266556. doi: 10.1371/journal.pone.0266556 (PMC9004753; doi:10.1371/journal.pone.0266556)
Supplement: S2 Table — Adjusted Rand Index of k-means clustering. Accentual-syllabic meters are systems of limitations superimposed on language. As such, they transform natural morphological and syntactical affordances [35]. This is why we can distinguish poetry from prose so easily based on word frequencies [5]. In addition, the distribution of parts of speech differs across metrical forms. Some words are more common in ternary meters simply because they are less likely to appear in binary meters for prosodic reasons. This has little connection with the semantics of meter but instead reflects the structural properties of verse. To make sure our pre-processing steps mitigated the problem of morphological differences, we repeat the clustering procedure from the H1 analysis. Here we use the frequencies of parts of speech that were included in LDA model (nouns, adjectives, verbs) as a feature set. S2 Table. shows that accuracy is visibly lower in this case than when clustering is topic-based. (PDF) [file pone.0266556.s010.pdf]

Czech		German		Russian		Dutch		English	
median	IQR	median	IQR	median	IQR	median	IQR	median	IQR
0.11	0.02	0.42	0.11	0.10	0.04	0.12	0.05	0.56	0.27
