# Supplementary material for: Semantics of European poetry is shaped by conservative forces: The relationship between poetic meter and meaning in accentual-syllabic verse
Source: PLoS One. 2022 Apr 12;17(4):e0266556. doi: 10.1371/journal.pone.0266556 (PMC9004753; doi:10.1371/journal.pone.0266556)
Supplement: S3 Table — The most distinctive topics for the most common meters. For each meter, topic probabilities are averaged across the entire corpus. These values are transformed into z-scores across particular meters. The table shows the five topics with the highest z-scores for each meter. (PDF) [file pone.0266556.s011.pdf]

Czech			
I4	I5	T4	T5
kniha, psát, verš	duch, síla, život	blaho, blahý, srdce	vlast, národ, český
ňadro, hruď, srdce	kniha, psát, verš	bůh, ctnost, svět	král, trůn, říše
jaro, jarní, květ	velký, věk, lidstvo	bůh, dík, Toba	pravit, dít, děva
vědět, povědět, dít	duše, cítit, srdce	dobrý, pan, říkat	pravda, slovo, řeč
rok, den, čas	tvář, zrak, oko	mladý, hoch, dívka	jiný, mnohý, svět
German			
I3	I4	I5	T4
Wald, Berg, Feld	rufen, sehen, kommen	können, kommen, sehen	Baum, Zweig, grün
Dan, welt, loben	laufen, tanzen, Kopf	leise, lauschen, stehen	Bruder, begraben, Muss
Kind, wär, arm	Mann, Weib, Frau	Flamme, Feuer, Glut	schön, Venus, zieren
Baum, Zweig, grün	sprechen, sagen, tun	Leben, Gefühl, Herz	Schönheit, hold, lieblich
werd, ehren, würd	gut, Geld, schlecht	hoch, Flügel, fliegen	einen, Einer, rasch
Russian			
I4	I5	I6	T4
vladyka, bog, gospod’	hram, venets, altar’	dar, dostojnyj, serdtse	knjaz’, rus’, vitjaz’
davat’, mysl’, slovo	den’, drug, moch’	roscha, holm, les	chasha, vino, pir
poet, pevets, muza	ljubov’, strast’, serdtse	uzhasnyj, strashnyj, smert’	deva, junyj, zhenih
nadezhda, dusha, zhizn’	dusha, chuvstvo, serdtse	pravo, chin, primer	ruchej, krylo, ptitsa
vdohnovenie, vostorg, mechta	vdohnovenie, vostorg, mechta	pravda, dobro, zlo	zoloto, stena, pyshnyj
Dutch			
I3	I4	I5	T4
lief, mogen, pijn	iesu, jesu, christum	dij, all, as	min, zin, hebben
zeer, hebben, doen	vader, geven, geest	heilig, heiligen, geest	wijn, drinken, bier
gods, woord, worden	bloed, vlees, ziel	heer, hebben, zullen	zult, zullen, geven
moeten, tijd, mogen	sijt, beminde, getal	end, wt, zullen	zoet, kus, kussen
satan, sint, godes	kind, moeder, klein	du, mit, dij	vrolijk, laten, hond
English			
I4	I5		
knight, lord, hall	sick, find, whole		
harp, strain, bard	new, good, old		
spear, horse, steed	great, tree, small		
roof, tower, pile	mind, kind, hail		
word, speak, answer	certain, blame, glide		
