# Supplementary material for: Semantics of European poetry is shaped by conservative forces: The relationship between poetic meter and meaning in accentual-syllabic verse
Source: PLoS One. 2022 Apr 12;17(4):e0266556. doi: 10.1371/journal.pone.0266556 (PMC9004753; doi:10.1371/journal.pone.0266556)
Supplement: S4 Table — Step 1 gives the number of poems after exclusion of other than accentual-syllabic poems (these include for instance free verse, accentual verse, syllabic verse), poems where less than 80% of lines are written in a single meter, poems outside the selected time span and poems outside the required length span (4 to 100 lines). The figure in parentheses gives how many poems remain after this step as compared to the entire corpus. Step 2 gives the number of poems when keeping only the most common iambic and trochaic meters. The figure in parentheses gives how many poems remain after this step as compared to the previous one. (PDF) [file pone.0266556.s012.pdf]

	Czech	German	Russian	Dutch	English
Step 1	39594 (57%)	11340 (21%)	11769 (66%)	8011 (36%)	3254 (50%)
Step 2	37097 (94%)	9910 (87%)	8972 (76%)	7366 (92%)	2977 (91%)
