# Supplementary material for: Semantics of European poetry is shaped by conservative forces: The relationship between poetic meter and meaning in accentual-syllabic verse
Source: PLoS One. 2022 Apr 12;17(4):e0266556. doi: 10.1371/journal.pone.0266556 (PMC9004753; doi:10.1371/journal.pone.0266556)
Supplement: S5 Table — Fit of a Bayesian regression model with Hamiltonian Monte Carlo sampling (4 chains, 2000 iterations, 1000 warmup); brms formula: ARI|trunc(up = 1) ∼ 1 + period * language. Categories are index-coded, reference level for predictors is Czech for languages and early for period. Bulk_ESS and Tail_ESS are measures of effective sample size. at 1 signifies chain convergence. effective sample size. R^ at 1 signifies chain convergence. (PDF) [file pone.0266556.s013.pdf]

	Estimate	Estimated Error	Low 95% CI	Up 95% CI	$\hat{R}$	Bulk_ESS	Tail_ESS
Intercept	1.47	0.05	1.38	1.56	1	2029	2387
period_late	-0.44	0.04	-0.52	-0.35	1	2153	2502
lang_de	-0.13	0.05	-0.23	-0.02	1	2427	2402
lang_ru	-0.7	0.04	-0.78	-0.61	1	2030	2482
period_late:lang_de	-0.47	0.06	-0.58	-0.35	1	2136	2639
period_late:lang_ru	0.29	0.05	0.2	0.39	1	1948	2403
